# Generation of cytotoxic T cell responses to an HLA-A24 restricted epitope peptide derived from wild-type *p53*

**DOI:** 10.1054/bjoc.2000.1715

**Published:** 2001-04

**Authors:** Y Umano, T Tsunoda, H Tanaka, K Matsuda, H Yamaue, H Tanimura

**Affiliations:** Second Department of Surgery, Wakayama Medical School; Department of Surgery and Bioengineering, Advanced Clinical Research Center, Institute of Medical Science, The University of Tokyo

**Keywords:** p53, CTL, HLA-A24, peptide

## Abstract

Mutations in the *p53* gene are the most common genetic alterations found in human tumours, and these mutations result in high levels of p53 protein in the tumour cells. Since the expression levels of wild-type p53 in nonmalignant tissue are usually much lower in contrast, the p53 protein is an attractive target for cancer immunotherapy. We tested p53 encoded HLA-A24 binding peptides for their capacity to elicit anti-tumour cytotoxic T lymphocytes (CTL) in vitro. These peptides were in murine p53-derived cytotoxic peptides, which were being presented to CTL by H-2K _d_ and H-2K _b_ molecules, because the HLA-A24 peptide binding motifs were similar to the H-2K _d_ and H-2K _b_. For CTL induction, we used CD8^+^T lymphocytes from the peripheral blood mononuclear cells (PBMC) of healthy donors and the peptides from pulsed dendritic cells as antigen-presenting cells. We identified the peptide, p53-161 (AIYKQSQHM), which was capable of eliciting CTL lines that lysed tumour cells expressing HLA-A24 and p53. The effectors lysed C1RA24 cells (p53^+^, HLA-A*2402 transfectant), but not their parental cell lines C1R (p53^+^, HLA-A,B null cell). These results strongly indicate that the CTL exerts cytotoxic activity in HLA-A24's restricted manner. The identification of this novel p53 epitope for CTL offers the possibility to design and develop specific immunotherapeutic approaches for treating tumours with p53 mutation in HLA-A24-positive patients. © 2001 Cancer Research Campaign http://www.bjcancer.com


*British Journal of Cancer* (2001) **84**(8), 1052–1057
© 2001 Cancer Research Campaign
doi: 10.1054/ bjoc.2001.1715, available online at http://www.idealibrary.com on IDEAL

### Generation of cytotoxic T cell responses to an HLA-A24 restricted epitope peptide derived from wild-type *p53*

**Y Umano[1], T Tsunoda[1,2], H Tanaka[1], K Matsuda[1], H Yamaue[1] and H Tanimura[1]**

[1]Second Department of Surgery, Wakayama Medical School; [2]Department of Surgery and Bioengineering, Advanced Clinical Research Center, Institute of Medical Science, The University of Tokyo

**Summary** Mutations in the *p53* gene are the most common genetic alterations found in human tumours, and these mutations result in high levels of p53 protein in the tumour cells. Since the expression levels of wild-type p53 in nonmalignant tissue are usually much lower in contrast, the p53 protein is an attractive target for cancer immunotherapy. We tested p53 encoded HLA-A24 binding peptides for their capacity to elicit anti-tumour cytotoxic T lymphocytes (CTL) in vitro. These peptides were in murine p53-derived cytotoxic peptides, which were being presented to CTL by H-$2K_d$ and H-$2K_b$ molecules, because the HLA-A24 peptide binding motifs were similar to the H-$2K_d$ and H-$2K_b$. For CTL induction, we used $CD8^+$ T lymphocytes from the peripheral blood mononuclear cells (PBMC) of healthy donors and the peptides from pulsed dendritic cells as antigen-presenting cells. We identified the peptide, p53-161 (AIYKQSQHM), which was capable of eliciting CTL lines that lysed tumour cells expressing HLA-A24 and p53. The effectors lysed C1RA24 cells ($p53^+$, HLA-A*2402 transfectant), but not their parental cell lines C1R ($p53^+$, HLA-A,B null cell). These results strongly indicate that the CTL exerts cytotoxic activity in HLA-A24's restricted manner. The identification of this novel p53 epitope for CTL offers the possibility to design and develop specific immunotherapeutic approaches for treating tumours with p53 mutation in HLA-A24-positive patients. © 2001 Cancer Research Campaign http://www.bjcancer.com

**Keywords**: p53; CTL; HLA-A24; peptide

Several clinical trials aimed at the induction of antitumour reactivity via vaccination against tumour-associated antigens, gp100, MAGE3, CEA, are ongoing (Marchand et al, 1995; Rosenberg et al, 1998; Morse et al, 1999). It is very important to search for tumour antigens which are widely expressed targets for CTLs restricted to major histocompatibility complex (MHC) class I for the development of T cell-mediated immunotherapy of cancer (Vierboom et al, 1997). The gastrointestinal adenocarcinomas are very common and a number of patients die of various kinds of adenocarcinomas. Therefore, the antigens expressed in adenocarcinomas are expected targets for immunotherapy. The p53 is expressed in tumour cells of 50% of patients with cancer (Harris, 1996). Regarding gastro-intestinal adenocarcinoma, the p53 expression was shown to be relatively high compared with that observed in other tumours. p53 overexpression is observed in 62% of oesophageal squamous cell carcinoma, 50–60% of gastric carcinoma, 55% of colon carcinoma, 50–60% of pancreatic carcinoma, and 58% of gall bladder carcinoma (Campo et al, 1991; Wever, 1998). It has been clarified that p53 serves as a target for immunorecognition and in fact, Deleo et al initially identified p53 as a tumour antigen (Deleo et al, 1979). Recently, p53 was identified as a tumour antigen and p53 epitope peptides as suitable for presentation by HLA molecules (Yanuck et al, 1993; Theobold et al, 1995; Mayordomo et al, 1996; Theobald et al, 1997; Gnjatic et al, 1998; Chikamatsu et al, 1999). These immunoresponses are generally linked to the mutations of the p53 gene that result in the accumulation of dysfunctional p53 product in the nucleus or cytoplasm of tumour cells (Ropke et al, 1996; Roth et al, 1996; Yu et al, 1997; McCarty et al, 1998; Theobald et al, 1998; Schawarz et al, 1999). Since the defects in overexpressed p53 are mostly caused by point mutations, the majority of the wild-type epitopes appeared to be conserved. Therefore, the overexpressed and non-mutated epitopes could be the immunotherapeutic targets for cytolytic response in most of the patient population.

*Received 26 June 2000*
*Revised 2 January 2001*
*Accepted 11 January 2001*

*Correspondence to:* T Tsunoda

The HLA-A2 restricted CTL-inducing peptides have been reported in the non-mutated region of wild-type p53. Todd et al reported that HLA-A*0201 restricted murine CTL lines specific to epitope 149–157 of p53 suppressed the growth of established tumours in vivo (Yanuck et al, 1993). Gnjatic et al showed that CTL lines recognizing p53 epitope 264–272 kill the breast carcinoma cells and the melanoma cells displaying the accumulated p53 protein (Gnjatic et al, 1998). However, the tumour rejection peptides that are restricted by HLA-A24 have not been identified yet. Since HLA-A24 is one of the most popular HLA alleles in Japanese (>60%) and also in Caucasians (Kubo et al, 1994), it appears to be important to find such peptides if it exists.

CTL epitopes from numerous TAA have been identified by multiple methods (Celis et al, 1994). In this study, p53-derived cytotoxic peptides were predicted from murine p53-derived cytotoxic peptides which were presented to CTL by H-$2K_d$ and H-$2K_b$ molecules, since binding motifs of the HLA-A24 peptide are similar to those of H-$2K_d$ and H-$2K_b$. We predicted 3 peptides, and then tested their capacity to bind to HLA-A24 molecules. This study shows that one of them can induce tumour reactive CTL responses in vitro from PBMC of HLA-A24 positive healthy volunteers in vitro.

### MATERIALS AND METHODS

### Cell lines

The TISI cells, human B lymphoblastoid cell lines expressing HLA-A*2402, were used for peptide-mediated cytotoxic assays.

The CIR cells are HLA-A,B null cells (HLA-C; weakly positive). The C1R-A24 cells are the C1R cells transfected with HLA-A*2402 gene. The C1R and C1R-A24 cells were kindly provided by Dr Takiguchi (University of Kumamoto, Japan). HT29(HLA-A24/1), colon carcinoma cell line, and NKN45(HLA-A24/24), gastric carcinoma cell line, were provided by Takara Shuzo Co, Ltd. (Otsu, Japan). PC9 (HLA-A24/2), lung adenocarcinoma cell line was kindly provided by Dr Yasumoto (University of Occupational and Environmental Health, Japan). These cell lines were maintained in a tissue cultureflask using RPMI 1640 supplemented with HEPES, antibiotics, and 10% heat-inactivated fetal bovine serum (FBS) (Gibco, BRL). The accumulation of p53 in all the cell lines in this study was analysed using immunohistochemistry. The p53 was accumulated in HT29, PC9 and C1R-A24 cells. However, expression of p53 was not detected in MKN45, and TISI cells. The mutation-analysis of p53 (exons 5–8) was performed on HT29, MKN45, PC9, C1R-A24 and TISI cell lines using single strand conformation polymorphism (SSCP) and direct sequence analysis. These results are summarized in Table 1.

### Synthesis of *p53*-derived peptides

Peptides were synthesized according to the standard solid phase synthesis method and purified by reversed phase high performance liquid chromatography (HPLC). The purity (>97%) and the identity of the peptides were determined by analytical HPLC and mass spectrometry analysis, respectively. Peptides were dissolved in dimetilsulphoxide (DMSO) at 20 mg ml$^{-1}$ and stored at –30˚C.

### MHC binding assay by flowcytometry

The binding affinity of the peptides for HLA-A24 molecules was examined using C1R-A24 cells. A total of $1 \times 10^6$ cells were washed 2 times in phosphate buffered saline (PBS) to remove contaminating calf serum proteins. Peptides binding to HLA-A24 molecules on C1R-A24 were stripped by mild acid treatment with an ice cold citrate phosphate buffer (0.131 M citric acid and 0.066 M $Na_2HPO_4$) (Stomkus et al, 1993). The buffer was adjusted to pH 3.3 with 5N NaOH or 5N HCl; this pH is essential for optimal elution of bound peptides and reconstitution of MHC class 1 molecule with the exogenously added peptides. Two millilitres of citrate phosphate buffer were added, and cell pellets were resuspended with gentle hand rocking for 60 s. Immediately after incubation, the eluted cells were buffered with a cold serum-free medium, and washed twice. Residual medium was removed by aspiration after the wash. To load the synthetic peptides, the C1R-A24 cells were incubated with 20 μg ml$^{-1}$ of synthetic peptides for 2 h at 4˚C. After the washing, the expression levels of MHC class 1 molecules were determined by flowcytometric analysis using an anti class 1 antibody (W6/32) (Immunotec, Marseille, France).

**Table 1** Human cell lines in this study

| Cells | HLA–A24 Expression[1] | p53 status[2] | p 53 Accumulation[3] |
|---|---|---|---|
| TISI | + | WT[4] | – |
| C1R | – | pmR273 to H[5] | + |
| C1RA24 | + | pmR273 to H | + |
| PC9 | + | pmR248 to Q | + |
| HT 29 | + | pmR 273 to H | + |
| MKN 45 | + | WT | – |

[1]HLA–A24 expression was determined serologically.
[2]Based on sequence analysis of reverse transcription PCR products corresponding to exons 5~8.
[3]p53 protein overexpression was estimated by immunocytochemistry.
[4]WT, wild type
[5]pm, point mutation

### Primary CTL induction cultures

Monocyte-derived DCs were used as antigen-presenting cells (APC) to trigger CTL responses using an MHC binding peptide. DCs were generated in vitro as described previously (Romani et al, 1994) (Romani and Gruner, 1994; Nukaya et al, 1999). Briefly, PBMCs isolated from a normal volunteer (HLA-A24) by Ficoll-Paque (Pharmacia, Piscataway, NJ) were separated by adherence to a plastic tissue culture flask to enrich the monocyte fraction. The monocyte-enriched population was then cultured in the presence of 1000 U ml$^{-1}$ of granulocyte macrophage colony stimulating factor (GM-CSF) (Genzyme) and 2000 U ml$^{-1}$ IL-4 (Genzyme) in RPMI 1640 containing HEPES and supplemented with 2 mM of L-glutamine, 1 mM of sodium pyruvate and 0.1 mM of non- essential amino acid solution, 5% heat–inactivated AB human serum (HS) (all reagents from BioWhittakar, Walkersville, MD), and antibiotics (RPMI/5% HS). After 7 days in the culture, generated DC was pulsed with 40 μg ml$^{-1}$ of MHC-binding peptides in the presence of 3 μg ml$^{-1}$ of $\beta_2$-microglobulin for 4 h at 20˚C in phosphate-buffered saline (PBS) containing 1% bovine serum albumin. The peptide-pulsed DC was then irradiated (5500 rad) and mixed at a 1:20 ratio with autologous $CD8^+$ T cells, obtained by positive selection using Dynabeads M-450 CD8 (Dynal, Lake Success, NY) and Detachabead (Dynal) following manufacturer's protocol. These cultures were set up in 48-well plates; each well contained $0.25 \times 10^5$ peptide-pulsed DC, $5 \times 10^5$ $CD8^+$ cells and 10 ng ml$^{-1}$ IL-7 (Genzyme) in 0.5 ml of RPMI/5% HS. One day later, the CTL cultures were supplemented with IL-10 (R&D Systems, Minneapolis, MN) to a final concentration of 10 ng ml$^{-1}$. On days 7 and 14, the T cell cultures were restimulated with autologous peptide-pulsed adherent APC. To prepare the adherent APC, $2 \times 10^6$ irradiated autologous PBMC in 0.5 ml of RPMI/5% HS was added to each well of 48-well plates. After incubation at 37˚C for 90 min, the non-adherent cells were washed off, and the adherent cells were incubated for 2 h with 20 μg ml$^{-1}$ of peptide and 3 μg ml$^{-1}$ $\beta_2$- microglobulin in a final volume of 0.25 ml of RPMI/5% HS per well. Supernatants of the responder cultures were aspirated and RPMI/5% HS was added to the total volume of 0.5 ml well$^{-1}$. After removing the un-loaded peptide from the wells of adherent APC, the responder cultures were transferred to the corresponding wells containing peptide-pulsed APC. Each well was restimulated separately and on the day following each restimulation, a final concentration of 10 ng ml$^{-1}$ of IL-10 was added to each culture. The cultures were fed every 2–3 days with RPMI/5% HS containing 10 U ml$^{-1}$ of IL-2 (Shionogi, Osaka, Japan). Cytotoxic activity was tested after 2 and 4 rounds of peptide stimulation on days 21 and 36 using peptide-pulsed TISI cells. Responder cells in positive wells for peptide killing were expanded as described below and tested for their cytotoxity.

### CTL assay

The tumour cells used as targets in the cytotoxicity assays were cultured as described previously in the presence of IFN–γ (100 U ml$^{-1}$) to enhance the expression of MHC class 1 molecules. The TISI

© 2001 Cancer Research Campaign cells were not treated. Adherent target cells were detached from tissue culture flasks with trypsin-EDTA. All cells were labelled with 200 μ Ci of $^{51}$Cr (Daiichi Kagaku Yakuhin, Tokyo, Japan) per 3 × $10^6$ cells for 1 h at 37˚C. Peptide-pulsed targets were prepared by incubating the cells at 5 × $10^5$ cells ml$^{-1}$ with 10 μg ml$^{-1}$ of the peptide for 16 h at 37˚C. Target cells were washed by centrifugation and mixed with effectors in a final volume of 0.2 ml in round-bottom microtitre plates. The plates were centrifuged (2 min at 400 g) to increase cell-to-cell contact and placed in a $CO_2$ incubator at 37˚C. After 4 h of incubation, 0.1 ml of the supernatant was collected from each well and radioactivity was determined in a gamma counter. To minimize non-specific lysis due to NK-like effectors, the 10–30 fold excess of unlabelled K562 cells were added. The MHC restriction was examined by testing the inhibition of the cytotoxicity by anti HLA-class I MAbs W6/32 and anti HLA-class II MAbs L243 and anti CD8 (Immunotec, Marseille, France). Cold target inhibition experiments utilized using unlabelled C1R cells and C1RA24 cells for completion for the recognition of $^{51}$Cr-labelled HT29 tumour cells.

The percentage of specific cytotoxicity was determined by calculating the percentage of specific $^{51}$Cr-release by the following formula: [(cpm of the test sample release – cpm of the spontaneous release)/(cpm of the maximum release – cpm of the spontaneous release)] × 100. The spontaneous release was determined by incubating the target cells alone in the absence of effectors, and the maximum release was obtained by incubating the targets with 1 N HCl. All determinants were done in triplicate, and the standard deviation error was consistently below 10% of the mean value (Nukaya et al, 1999).

### Procedure of CTL expansion

To obtain sufficient cell numbers for detailed characterization, CTL lines were expanded in tissue cultures following methods similar to the ones described by Walter et al (Walter, 1995; Salgauer et al, 1996; Nukaya et al, 1999). A total of 5 × $10^4$ CTL were resuspended in 25 ml of RPMI/5% HS with 25 × $10^6$ irradiated (3300 rads) PBMC and 5 × $10^6$ irradiated (8000 rads) Epstein–Barr virus-transformed β-lymphoblastoid cell line EHM cells (HLA-A3/3) in the presence of 30 ng ml$^{-1}$ of anti-CD3 MAb. One day after initiating the cultures, 120 IU ml$^{-1}$ of IL-2 was added to the cultures. The cultures were fed with fresh RPMI/5% HS containing 30 IU ml$^{-1}$ of IL-2 on days 5, 8 and 11 and were split if the T-cell concentration reached numbers >1.5 × $10^6$ ml$^{-1}$. On average, approximately 1–2 × $10^7$ CTLs were obtained by days 12–14.

### RESULTS

### HLA-A24 binding peptides from the murine p53 cytotoxic peptides

To identify the potential epitope peptides of murine p53, the sequence of murine p53 were examined for the presence of specific MHC binding motifs for the H-2K$_d$ and H-2K$_b$ as examined for specific HLA-A alleles (Celis et al, 1994). 3 peptides were selected based on the high-binding motifs as shown in Table 2, that is, p53-137 (amino acids, 137-148) (Yaruck et al, 1993), p53-161 (161-169) (Vierboom et al, 1997), and p53–235 (235-243) (Mayordomo et al, 1996). These peptides were synthesized and their binding affinities were analysed by HLA-A24 peptide-binding assay, using C1R-A24 cells.

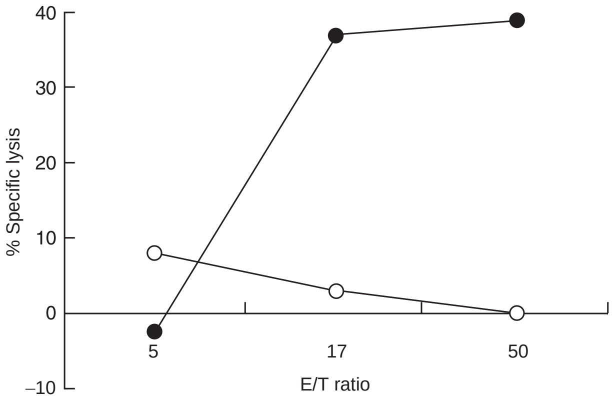

**Figure 1** Cytotoxic activity of CTL against peptide pulsed target cells. The CTL line (p53-161-41) expanded after 4 cycles of restimulations with peptides were tested for their cytotoxic activity. The recognition of peptide (p53-161) pulsed TISI by the CTL. ●, TISI pulsed with peptide (p53-161); ○, TISI without peptide

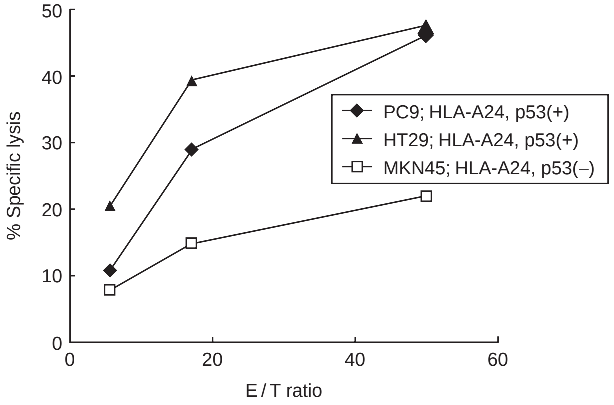

**Figure 2** Cytotoxic activity against the various tumour cell lines by the CTL line (p53-161-41). The cytotoxic activity of the effectors was assessed against HT29 (colon carcinoma cell line, HLA-A24+, P53+), PC9 (lung adenocarcinoma cell line, HLA-A24+, p53+), MKN45 (gastric carcinoma cell line, HLA-A24+, P53-), at various E/T ratios

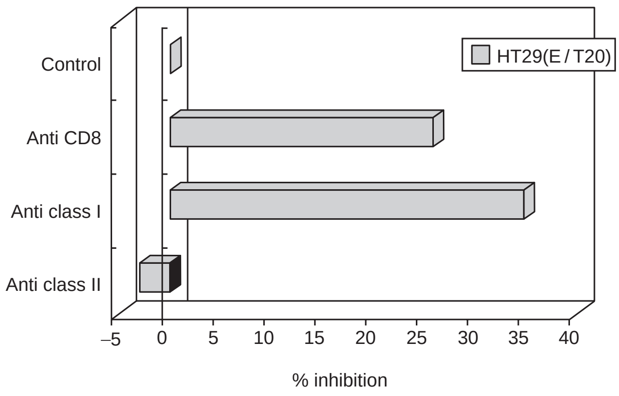

**Figure 3** Inhibition of specific cytotoxic activity of the CTL line (p53-161-41) by MAbs. $^{51}$Cr labelled HT29 tumour cells (HLA-A24+, p53+) were preincubated with anti-HLA class I MAb or anti-HLA class II MAb, or the CTLs were preincubated with anti-CD8 MAb for 1 h at room temperature. After the incubations, effectors and targets were mixed at an E/T ratio of 20. The cytotoxic activity was determined after 4 h incubation at 37˚C

*© 2001 Cancer Research Campaign*

**Table 2** Peptide prediction from murine p53 to human p53

| Murine p53 | Human p53 |
|---|---|
| 134–145 LAKTCPVQLWVS | 137–148 X‡XXXXXXXXXXD (p53–137) |
| 158–166 AIYKKSQHM | 161–169 XXXQXXXX (p53–161) |
| 232–240 KYMCNSSCM | 235–243 NXXXXXXXX (p53–235) |

X*: same amino acids between murine p53 and human p53.

The C1R-A24 cell was incubated in the presence of 20 μg ml$^{-1}$ of peptide for 2 h, stained with W6/32, the HLA class I specific antibody, and analysed with flowcytometry. Expression level of the MHC class I molecules was significantly higher than the negative control when p53-137 or p53-161 was pulsed (142 of mean channels in the p53-161 peptide pulsed cells, 115 in the p53-137, whereas 100 in the non peptide-pulsed cells). These results indicate that 2 predicted peptides had HLA-A24 binding affinities.

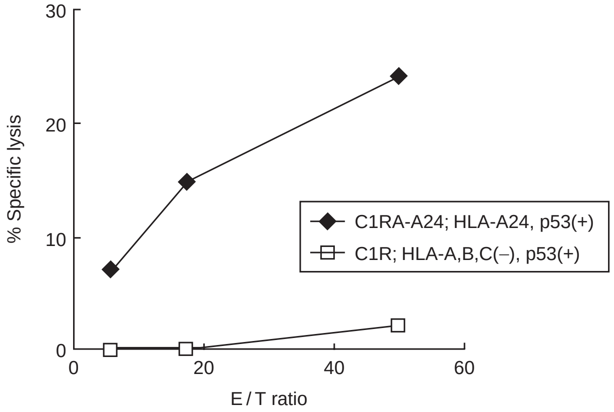

**Figure 4** HLA-A*2402 restricted cytotoxic activity by the CTL line (p53-161-41). The cytotoxicity of the CTL was tested by 4 h CRA at various E/T ratios. Target cells were C1R (p53+, HLA-A,B,C null cell), and C1R-A24 (p53+, HLA-A*2402 transfectant)

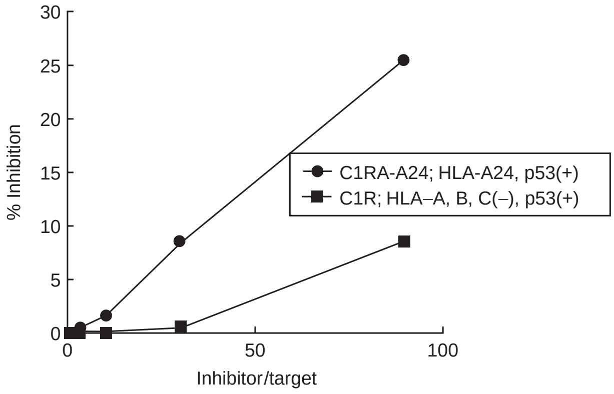

**Figure 5** Cold target inhibition assay with CTL line (p53-161-41) at 50 of E/T ratio. Hot target was HT29 (p53+, HLA-A*2402). Cold targets were C1R (p53+, HLA-A,B,C negative) or C1RA24 (p53+, HLA-A*2402)

### CTL induction

The p53-161 and p53-137 peptides were studied for their capacity to induce CTL in vitro. To examine this question, PBMCs from 6 HLA-A24$^+$ healthy donors were stimulated using DCs pulsed with synthetic peptides. The p53-161 peptide induced tumour-specific CTL in 2 of the 4 donors. However, the p53-137 peptide did not induce tumour-specific CTL. After 4 rounds of restimulation, these CTLs that lysed peptide–pulsed target cells were expanded using anti-CD3 MAb and a mixture of feeder cells for further analysis. Peptide specific CTL responses were measured in a 4 h $^{51}$Cr releasing assay (CRA) using peptide-pulsed TISI cells as targets. As shown in Figure 1, the CTL raised against p53-161 (line 41) efficiently lysed TISI cells only when they were pulsed with p53-161 peptide. Furthermore, the p53-specific CTL lines lysed the various cell lines which have p53 overexpression with HLA-A24. The tumour lines, pretreated with 100 U ml$^{-1}$ of IFNγ for 48 h, and non-labelled (cold) K562 cells were added to target populations. HT29 (colon carcinoma, p53$^+$, HLA-A24$^+$), PC9 (lung adenocarcinoma, p53$^+$, HLA-A24$^+$) cells were significantly lysed by the bulk CTL line 41, but MKN45 (gastric carcinoma, p53$^-$, HLA-A24$^+$) cells were scarcely lysed (Figure 2).

### Characterization of the established CTLs

To examine the characteristics of CTL lines raised against p53 peptide, MAbs against HLA class I MAb, HLA class II MAb, CD8 were tested for their capacity to inhibit the cytotoxic activity. The cytotoxicity of CTL against the HT29 targets was significantly reduced when anti class I and anti CD8 MAb were used, indicating that the CTL lines recognize the p53 derived peptide in an HLA class I restricted manner (Figure 3). Furthermore, the CTL lysed C1R-A24 (p53$^+$, HLA-A*2402 transfectant) cells, but not their parent cell lines C1R (p53$^+$, HLA A, B, C null cell) (Figure 4). These results clearly indicate that the cytotoxic activity of the CTLs is HLA-A*2402 in a restricted manner. Cold target inhibition experiments were performed using unlabelled C1R cells and C1R-A24 cells to examine the specificity of CTL recognizing HT29 tumour cells. The cytotoxicity against the HT29 cells was suppressed with the cold C1R-A24 cells but not with C1R cells (Figure 5). Thus, these results strongly suggest that the CTL lines raised against p53-161 recognize p53-derived epitopes in HLA-A*2402 restricted fashion.

### DISCUSSION

Tumour antigens, which are specifically presented by a class I MHC molecules on the multiple types of tumours, are very good candidates for cancer vaccine (Harris, 1996). However, most of the TAAs currently available are expressed on specific tumour types (Salgaller et al, 1996; Lee et al, 1999), and most of the epitope peptides identified are restricted to HLA-A2 allele (Butterfield et al, 1999; Vissers et al, 1999). This situation limits the broad applicability of this approach. To improve the situation, efforts have been made to identify TAAs expressed in multiple cell types (van den Burg et al, 1995; Tanaka et al, 1997; Fujie et al, 1999; Kikuchi et al, 1999; Oiso et al, 1999). As the same time, new tumour antigenic peptides restricted to other HLA class I alleles have been explored (van den Burg et al, 1996; Nestle et al, 1998; Maric and Liu, 1999; Wang et al, 1999; Yang et al, 1999).

© 2001 Cancer Research Campaign 

Mutant p53 is expressed in 50% of malignant tumours. Especially gastrointestinal carcinomas, which are the common cancers in the Asian region, mutation of p53 is observed more frequently when compared with that observed in tumours of other types. The spectrum of p53 mutations is very wide and variable (Harris, 1996). However, most mutation in p53 is a single base missense-mutation confirmed in specific regions. Thus, some of the wild-type epitopes outside of the area are maintained in many cases. Wild-type p53 may be ignored by the immune system due to insufficient amounts and extremely short $t_{1/2}$ (Gnjatic et al, 1998). Mutated p53 would become immunogenic due to the stabilization and the accumulation in tumour cells. Therefore, the epitopes found in the conserved area of the wild-type p53 could serve as immunologic targets in various tumour types.

The idea that p53 can serve as a target for immunotherapy has been explored in the past (Zhou et al, 1999; Hernandez et al, 2000), and antigenic p53 peptides restricted by HLA-A2 have been described. However, the antigenic peptides restricted to HLA-A24 allele, most of which (90%) is HLA-A*2402 subtype and frequency (60%) expressed in Asians including Japanese (Nukaya et al, 1999). Thus, we have tried to identify a new p53 epitope peptide presented by HLA-A24.

In this study, we obtained the evidence that p53-161, nonamer peptide derived from wild-type p53, induces CTL in vitro. The induced CTL line showed specific cytotoxicity not only against the peptide-pulsed target cells but also against HLA-A24-positive adenocarcinoma cell lines overexpressing p53. Because the wild-type p53 is normally ignored by the immune system due to its short half-life, this response is not so much recognition of endogenously processed with wild-type *p53* in MKN45 cell or TISI cell as bulk CTL response. However, Ropke et al, reported that the accumulation of mutated p53 protein was not absolute requirement for killing by p53-specific CTL (Ropke et al, 1996). The relation between presentation of p53 epitope and recognition by p53 specific CTL still remains to be examined. Furthermore, these CTLs kill targets in an HLA-A24 restricted manner. These results suggest that the p53–161 peptide is one of p53 epitope peptides restricted to HLA-A24.

The P53–161 peptide (AIYKQSQHM) derived from human wild-type p53 was predicted from the epitope (AIYKKSQHM; amino acid 158–166) derived from mouse wild-type p53. The CTL, generated in p53-deficient mice and recognizing this peptide presented on H-$2K_b$ molecule was also capable of preventing the outgrowth of p53-overexpressed tumours in immunocompetent p53 (+/+) C57BL/6 mice (Vierboom et al, 1997). Typical HLA-A24 binding motifs have Tyr or Trp at position 2 and Phe, Leu or Ileu at position 9 of a nonamer peptide. Interestingly, p53-161 has Tyr at position 3, and Met, of which affinity is lower than Phe, Leu and Ileu, at position 9 (Kubo et al, 1994). These characteristics lead to low binding affinity of p53-161 peptide for HLA-A24 molecules. It is possible that other peptides with which high affinity could be expressed at high levels on the cell surface during initial T cell education may be deleted from the T-cell repertoire. Lower binding or subdominant determinants could have escaped this process for tolerance induction (van den Burg et al, 1995). Similar to some of the other peptide epitopes of TAAs (Solgaller et al, 1996), p53–161 has only one anchor residue and weak binding affinity to HLA-A24. It has been very difficult to treat the advanced and metastatic cancers with any exiting therapy including surgery, chemotherapy and radiotherapy. As the mutated p53 accumulates in most of the metastatic tumour cells, and is associated with the resistance to chemotherapy and radiotherapy, a p53-peptide-based immunotherapy could be helpful for patients with advanced cancer. Furthermore, immunization with a combination of other antigens including CEA and HER2/neu might lead to a successful outcome.

### ACKNOWLEDGEMENTS

We thank Dr Hideaki Tahara, Department of Surgery and Bioengineering, Advanced Clinical Research Center, Institute of Medical Science, The University of Tokyo for editing this manuscript. This work was supported in part by grants from the Ministry of Education, Science, Sports and Culture of Japan (No. 09671245).

### REFERENCES

Butterfield LH, Koh A, Meng W, Vollmer CM, Ribas A, Dissette V, Lee E, Glaspy JA, McBride WH and Economou JS (1999) Generation of human T cell responses to an HLA A2.1 restricted peptide epitope derived from α Fetoprotein. *Cancer Res* **59**: 3134–3142

Campo E, de la Calle Martin O, Miquel R, Palacin A, Romero M, Fabregat V, Vives J, Cardesa A and Yague J (1991) Loss of Heterozygosity of p53 gene and p53 protein expression in human colorectal carcinomas. *Cancer Res* **51**: 4436–4442

Celis E, Tsai V, Crimi C, Demars R, Wentworth PA, Chesnut RW, Grey HM, Sette A and Serra HM (1994) Induction of anti-tumor cytotoxic T lymphocytes in normal humans using primary cultures and synthetic peptide epitopes. *Proc Nat Acad Sci* **91**: 2105–2109

Chikamatsu K, Nakano K, Storkus WJ, Appella E, Lotze MT, Whiteside TL and DeLeo AB (1999) Generation of anti p53 cytotoxic T lymphocytes from human peripheral blood using autologous dendritic cells. *Clin Cancer Res* **5**: 1281–1288

DeLeo AB, Jay G, Appella E, Dubois DC, Low LW and Old LJ (1979) Identification of a transformation related protein in chemically induced sarcomas and other transformed cells of the mouse. *Proc Natl Acad Sci USA* **76**: 2420–2424

Fujie T, Tahara K, Tanaka F, Mori M, Takesako K and Akiyoshi T (1999) A MAGE 1 encoded HLA A24 binding synthetic peptide induces specific anti tumor cytotoxic T lymphocytes. *Int J Cancer* **80**: 169–172

Gnjatic S, Cai Z, Viguier M, Chouaib S, Guillet JG and Choppin J (1998) Accumulation of the p53 protein allows recognition by human CTL of a wild type p53 epitope presented by breast carcinomas and melanomas. *J Immunol* **160**: 328–333

Harris CC (1996) Structure and function of the p53 tumor suppressor gene: Clues for rational cancer therapeutic strategies. *J Natl Cancer Inst* **88**: 1442–1455

Hernandez J, Lee PP, Davis MM and Sherman LA (2000) The use of HLA A2.1/p53 peptide tetramers to visualize the impact of self tolerance on TCR repertoire. *J Immunol* **164**: 596–602

Kikuchi M, Nakao M, Inoue T, Matsunaga K, Shichijo S, Yamana H and Itoh K (1999) Identification of a SART 1 derived peptide capable of inducing HLA A24 restricted and tumor specific cytotoxic T lymphocytes. *Int J Cancer* **81**: 459–466

Kubo RT, Sette A, Grey HM, Appella E, Sakaguchi K, Zhu NZ, Arnott D, Sherman N, Shabanowitz J, Michel H, Bodnar WM, Davis TA and Hunt DF (1994) Definition of specific peptide motifs for four major HLA A alleles. *J Immunol* **152**: 3913–3924

Lee KH, Wang E, Nielsen MB, Wunderlich J, Migules S, Connors M, Steinberg SM, Rosenberg SA and Marincola FM (1999) Increased vaccine specific T cell frequency after peptide based vaccination correlates with increased susceptibility to in vitro stimulation but dose not lead to tumor regression *J Immunol* **163**: 6292–6300

Maric M and Liu Y (1994) Strong cytotoxic T Lymphocyte responses to a macrophage inflammatory protein 1 α expressing tumor: Linkage between inflammation and specific immunity. *Cancer Res* **59**: 5549–5553

Marchand M, Weynants P, Rankin E, Arienti F, Belli F, Parmiani G, Cascinelli N, Bourlond A, Vanwijck R and Boon T (1995) Tumor regression responses in melanoma patients treated with a peptide encoded by gene MAGE-3. *Int J Cancer* **63**: 883–885

Mayordomo JI, Loftus DJ, Sakamoto H, De Cesare CM, Appasamy PM, Lotze MT, Storkus WJ, Appella E and DeLeo AB (1996) Therapy of murine tumor with

 © 2001 Cancer Research Campaign p53 wild type and mutant sequence peptide based vaccines. *J Exp Med* **183**: 1357–1365

McCarty TM, Liu X, Sun J, Peralta EA, Diamond DJ and Ellenhorn JDI (1998) Targeting p53 for adoptive T cell immunotherapy. *Cancer Res* **58**: 2601–2605

Morse MA, Deng Y, Coleman D, Hull S, Fisher EK, Nair S, Schlom J and Ryback ME (1999) A phase 1 study of immunotherapy with Carcinoembryonic antigen peptide (CAP 1) pulsed, autologous human cultured dendritic cells in patients with metastatic malignancies expressing carcinoembryonic antigen. *Clin Cancer Res* **5**: 1331–1338

Nestle FO, Alijagic S, Gilliet M, Sun Y, Grabbe S, Dummer R, Burg G and Schadendorf D (1998) Vaccination of melanoma patients with peptide or tumor lysate pulsed dendritic cells. *Nature Med* **4**: 328–332

Nukaya I, Yasumoto M, Iwasaki T, Ideno M, Sette A, Celis E, Takesako K and Kato I (1999) Identification of HLA-A24 epitope peptide of carcinoembryonic antigen which induce tumor reactive cytotoxic T lymphocyte. *Int J Cancer* **80**: 92–97

Oiso M, Eura M, Katsura F, Takiguti M, Sobao Y, Masuyama K, Nakashima M, Itoh K and Ishikawa T (1999) A newly identified MAGE 3 derived epitope recognized by HLA A24 restricted cytotoxic T lymphocytes. *Int J Cancer* **81**: 387–394

Romani N and Gruner S (1994) Proliferating dendritic cell progenitors in human blood. *J Exp Med* **180**: 83–93

Ropke M, Hald J, Guldberg P, Zeuthen J, Norgaard L, Fugger L, Svejgaard A, van der Burg S, Nijman HW, Melief CJM and Claesson MH (1996) Spontaneous human squamous cell carcinomas are killed by a human cytotoxic T lymphocyte clone recognizing a wild type p53 derived peptide. *Proc Natl Acad Sci USA* **93**: 14704–14707

Rosenberg SA, Yang JC, Schwartzentruber DJ, Hwu P, Marincola FM, Topalian SL and White DE (1998) Immunologic and therapeutic evaluation of a synthetic peptide vaccine for the treatment of patients with metastatic melanoma. *Nature Medicine* **4**: 321–327

Roth J, Dittmer D, Rea D, Tartaglia J, Paoletti E and Levine AJ (1996) p53 as a target for cancer vaccines: Recombinant canarypox virus vectors exoressing p53 protect mice against lethal tumor. *Proc Natl Acad Sci USA* **93**: 4781–4786

Salgaller ML, Marincola FM, Cormier JN and Rosenberg SA (1996) Immunization against epitopes in the human melanoma antigen gp100 following patient immunization with synthetic peptides. *Cancer Res* **56**: 4749–4745

Schwarz RE, McCarty TM, Peralta EA, Diamond DJ and Ellenhorn JDI (1999) An orthotopic in vivo model of human pancreatic cancer. *Surgery* **126**: 562–567

Storkus WJ, Zeh III HJ, Salter RD and Lotze MT (1993) Identification of T cell epitopes: Rapid isolation of class I presented peptides from viable cells by mild acid elution. *J Immunother* **14**: 94–103

Tanaka F, Fujie T, Tahara K, Mori M, Takesako K, Sette A, Celis E and Akiyoshi T (1997) Induction of antitumor cytotoxic T lymphocytes with a MAGE 3 encoded synthetic peptide presented by human leukocytes antigen A24. *Cancer Res* **57**: 4465–4468

Theobald M, Biggs J, Dittmer D, Levine AJ and Sherman LA (1995) Tageting p53 as a general tumor antigen. *Proc Natl Acad Sci USA* **92**: 11993–11997

Theobald M, Biggs J, Hernandez J, Lastgarten J, Labadie C and Sherman LA (1997) Tolerance to p53 by A2.1 restricted cytotoxic T lymphocytes. *J Exp Med* **185**: 833–841

Theobald M, Ruppert T, Kuckelkorn U, Hernandez J, Haussler A, Ferreira EA, Koszinowski UH, Kloetzel PM and Sherman LA (1998) The sequence alteration associated with a mutational hotspot in p53 protects cells from lysis by cytotoxic T lymphocytes specific for a flanking peptide epitope. *J Exp Med* **188**: 1017–1028

van den Burg SH, Ras E, Drijfhout JW, Benckhuijsen WE, Bremers AJA, Melief CJM and Kast WM (1995) An HLA class 1 peptide binding assay based on competition for binding to class 1 molecules on intact human B cells, Identification of conserved HIV 1 Polymerase peptides binding to HLA A0301. *Hum Immunol* **44**: 189–198

van der Burg SH, Visseren MJW, Brandt RMP, Kast WM and Melief CJM (1996) Immunogenicity of peptides bound to MHC class I molecules depends on the MHC peptide complex stability. *J Immunol* **156**: 3308–3314

Vierboom MPM, Nijman HW, Offrnga R, van der Voort EIH, van Hall T, van den Broek L, Fleuren G, Kenemans P, Kast WM and Melief CJM (1997) Tumor eradication by wild type p53 specific cytotoxic T lymphocytes. *J Exp Med* **186**: 695–704

Vissers JLM, De Vries IJM, Schreurs MWJ, Engelen LPH, Oosterwijk E, Figdor CG and Adema GJ (1999) The renal cell carcinoma associated antigen G250 encodes a human leukocyte antigen HLA A2.1 restricted epitope recognized by cytotoxic T lymphocytes. *Cancer Res* **59**: 5554–5559

Walter EA (1995) Reconstitution of cellular immunity against cytomegalovirus in recipients of allogeneic bone marrow by transfer of T cell clones from the donor. *N Engl J Med* **333**: 1038–1044

Wang F, Bade E, Kuniyoshi C, Sppears L, Jeffery G, Marty V, Groshen S and Weber J (1999) Phase 1 trial of a MART 1 peptide vaccine with incomplete Freund's adjuvant for resected high risk melanoma. *Clin Cancer Res* **5**: 2756–2765

Weller M (1998) Predicting response to cancer chemotherapy: the role of p53, *Cell Tissue Res* **292**: 435–445

Yang D, Nakao M, Shichijo S, Sasatomi T, Takasu H, Mastumoto H, Mori K, Hayashi A, Ymana H, Shirouzu K and Itoh K (1999) Identification of a gene cording for a protein possessing shared tumor epitopes capable of inducing HLA–A24–restricted Cytotoxic T lymphocytes in cancer patients. *Cancer Res* **59**: 4056–4063

Yanuck M, Carbone DP, Pendleton CD, Tsukui T, Winter SF, Minna JD and Berzofsky JA (1993) A mutant p53 tumor suppressor protein is a target for peptide induced CD8+ cytotoxic T cells. *Cancer Res* **53**: 3257–3261

Yu Z, Liu X, McCarty TM, Diamond DJ and Ellenhorn JDI (1997) The use of transgenic mice to generate high affinity p53 specific cytolytic T cells. *J Surg Res* **69**: 337–343

Zhou X, Wong S, Walter J, Jacks T and Eisen HN (1999) Increased generation of CD8+ T cell clones in p53 mutant mice. *J Immunol* **162**: 3957–3960

*© 2001 Cancer Research Campaign*
